# Baicalin Augments 5-Fluorouracil Efficacy in Colorectal Cancer by Triggering MLKL-Dependent Necroptosis: A Novel Strategy to Overcome Chemoresistance

**DOI:** 10.3390/ijms27062919

**Published:** 2026-03-23

**Authors:** Jingwen Yuan, Zhiying Peng, Rongbo Wen, Leqi Zhou, Fuao Cao, Tianshuai Zhang, Yingjie Wu, Jiayue Wu, Ran Lin, Guanyu Yu, Wei Zhang

**Affiliations:** Colorectal Surgery Department, Changhai Hospital, Naval Medical University, Shanghai 200433, China; yuanjingwen718@whu.edu.cn (J.Y.); p1zy24@163.com (Z.P.); wrb1213@hotmail.com (R.W.); richard12@126.com (L.Z.); caofuao@163.com (F.C.); zhangtianshuai0106@163.com (T.Z.); smmuwyj@163.com (Y.W.); wujiayue03@163.com (J.W.); linr278@163.com (R.L.)

**Keywords:** baicalin, 5-fluorouracil, colorectal cancer, necroptosis, chemoresistance

## Abstract

5-Fluorouracil (5-Fu) remains essential in colorectal cancer (CRC) treatment, but monotherapy causes severe toxicity and faces chemoresistance. Combination regimens are encouraged to improve efficacy and safety. Natural compounds like Baicalin show anti-tumor potential in other gastrointestinal cancers, yet their role in CRC, particularly in overcoming 5-Fu resistance, is underexplored. The combined effect of Baicalin and 5-Fu was evaluated through in vitro functional assays and an in vivo xenograft model. Mechanisms were investigated using Western blot, qPCR, and RNA-seq. Baicalin enhanced 5-Fu to inhibit CRC progression both in vitro and in vivo. Mechanistically, Baicalin enhanced 5-Fu cytotoxicity by activating the MLKL-dependent necroptosis pathway. This study proposes the Baicalin and 5-Fu combination as a novel and potent chemosensitizing strategy for CRC, especially in 5-Fu-resistant cases, and provides a mechanistic rationale for Baicalin as a chemotherapy-enhancing agent.

## 1. Introduction

Colorectal cancer (CRC) remains a significant global health issue, being the third most frequently diagnosed cancer and the second highest cause of cancer-related mortality worldwide [1]. According to recent global cancer burden data, there were over 1.9 million new CRC cases and nearly 935,000 deaths in 2020, with incidence rates rising in rapidly developing countries. This alarming epidemiological trend underscores the urgent need for innovative therapeutic advancements. While surgical resection offers a potential cure for localized tumors, systemic chemotherapy serves as the cornerstone of treatment for patients with advanced or metastatic CRC, among whom the five-year survival rate remains dishearteningly low [2]. Currently, for metastatic CRC (mCRC), standard-of-care treatment has evolved to include combination regimens such as FOLFOX (5-Fu + Oxaliplatin) or FOLFIRI (5-Fu + Irinotecan), often in conjunction with targeted therapies like bevacizumab (anti-VEGF) or cetuximab (anti-EGFR) for select patient populations [3,4]. However, the efficacy of these regimens is ultimately limited by both primary and acquired resistance, highlighting the persistent need for novel, well-tolerated sensitizing agents.

5-fluorouracil (5-Fu), a fluoropyrimidine analog, has been a fundamental component of CRC chemotherapy for more than fifty years. Its primary mechanism of action involves inhibiting thymidylate synthase and incorporating it into RNA and DNA, thereby disrupting nucleic acid synthesis and inducing apoptotic cell death in rapidly proliferating cancer cells [5]. Despite its foundational role, the clinical application of 5-Fu is significantly hindered by a narrow therapeutic index and the frequent emergence of drug resistance [6]. Monotherapy with 5-Fu is particularly limited by a range of dose-limiting toxicities, including profound myelosuppression (neutropenia and thrombocytopenia), severe gastrointestinal damage (oral mucositis and diarrhea), and hand-foot syndrome. These adverse effects collectively impair patients’ quality of life and often necessitate dose reductions or treatment cessation [7,8], further compromising therapeutic outcomes. To mitigate these toxicities, research has focused on optimizing 5-Fu administration, including continuous infusion protocols and the development of oral prodrugs like capecitabine; however, these approaches do not fundamentally resolve the issue of inherent or acquired chemoresistance [8,9]. Consequently, the development of combination strategies aimed at enhancing 5-Fu efficacy while mitigating its adverse effects has become a central focus of modern CRC chemotherapy. This has resulted in the extensive use of 5-Fu together with agents such as oxaliplatin or irinotecan. However, these combinations often introduce their own unique and sometimes severe toxicities [10]. Furthermore, chemoresistance, particularly the acquired resistance to 5-Fu, continues to be a significant challenge in clinical settings. Thus, identifying novel, well-tolerated agents that can significantly enhanced 5-Fu to achieve “chemo-sensitization” and overcome drug resistance remains a highly pursued and clinically critical goal.

In this context, natural products derived from traditional medicinal plants have re-emerged as valuable sources of potential chemosensitizers, owing to their multi-targeted actions and generally favorable safety profiles [11,12]. Derived from the roots of *Scutellaria baicalensis* Georgi (Huang Qin), baicalin is a major bioactive flavonoid glycoside, it stands out as a promising candidate. For many years, this compound has been employed in traditional Chinese medicine to manage inflammatory conditions, and has garnered significant scientific attention due to its diverse pharmacological activities, including antioxidant, antiviral, and anticancer properties [13,14,15]. Baicalin has been shown to act through distinct pathways to delay the occurrence and progression of liver and gastric cancers [16,17], and its antitumor effects have also been reported in pancreatic and esophageal cancer models [18,19]. A particularly attractive feature of Baicalin for clinical translation is its well-established biocompatibility and low systemic toxicity, as demonstrated by numerous pharmacological and toxicological studies [20,21], making it an ideal candidate for combination chemotherapy. Crucially, Baicalin’s favorable safety profile, established in traditional use and modern pharmacological studies [20,21], coupled with its stability in biological systems and the low cost compare with other chemotherapy drugs [22], makes it an ideal candidate for chronic combination therapy. Its low cost and wide availability further enhance its translational potential, addressing key practical considerations for clinical adoption. Therefore, the rationale for combining baicalin with 5-Fu is not only based on potential mechanistic potentiation but also on the promising pharmaceutical properties of baicalin that could improve the therapeutic index of 5-Fu-based chemotherapy.

However, despite these promising observations in other cancer types, the specific role of Baicalin in CRC—especially its potential to enhance 5-Fu and reverse chemoresistance, as well as the precise molecular mechanisms underlying such interactions—remains inadequately explored, representing a significant knowledge gap. Elucidating this potential holds considerable translational value, given the urgent need to address 5-Fu resistance in clinical practice. The objective of the current study was to methodically analyze the combined impact of Baicalin and 5-Fu in CRC models. Employing an integrated approach that included in vitro functional assays, transcriptomic profiling, and in vivo validation in a xenograft model, we obtained compelling evidence of a potent combination effect interaction between Baicalin and 5-Fu. Most importantly, we mechanistically identified that This enhancement is chiefly mediated by the activation of the necroptosis pathway that relies on MLKL, a form of programmed necrosis. This novel insight not only deepens our understanding of Baicalin’s antitumor mechanism but also provides a robust scientific rationale for developing Baicalin as a promising adjuvant to 5-Fu-based chemotherapy, particularly for overcoming 5-Fu resistance in CRC.

## 2. Results

### 2.1. Cytotoxic Effects of Baicalin and 5-Fu on CRC Cells

**Figure 1:**
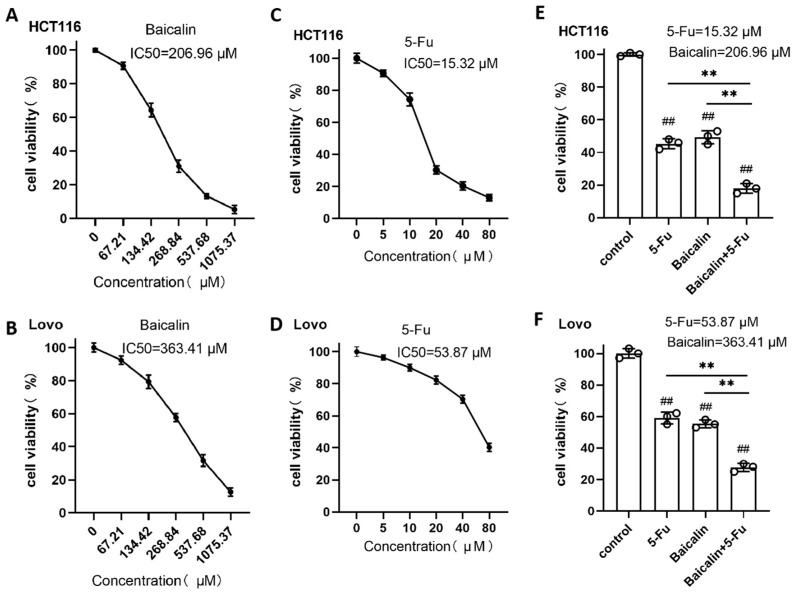
CCK-8 assay for determining drug IC_50_ in colorectal cancer cells. (**A**) Viability of HCT116 cells exposed to increasing concentrations of Baicalin; (**B**) Viability of Lovo cells treated with escalating doses of Baicalin; (**C**) Viability of HCT116 cells following treatment with different concentrations of 5-Fu; (**D**) Viability of Lovo cells incubated with varying concentrations of 5-Fu; (**E**) Viability of HCT116 cells after combined administration of Baicalin and 5-Fu; (**F**) Viability of Lovo cells subjected to the combination of Baicalin and 5-Fu. Data are presented as mean ± SEM from three independent biological replicates (n = 3). ns *p* > 0.05, # *p* < 0.05, ## *p* < 0.01 compared to the control group; ns *p* > 0.05, * *p* < 0.05, ** *p* < 0.01, *** *p* < 0.001 for comparisons between treatment groups.

We first evaluated the sensitivity of two human CRC cell lines, HCT116 and Lovo, to Baicalin and 5-Fu. A series of five graded concentrations was prepared for each drug (Supplementary Table S1). CCK-8 assays demonstrated that both agents inhibited the viability of CRC cells in a concentration-dependent manner. The calculated half-maximal inhibitory concentration (IC_50_) of Baicalin was 206.96 μM for HCT116 cells (Figure 1A) and 363.41 μM for Lovo cells (Figure 1B). The IC_50_ values of 5-Fu were 15.32 μM in HCT116 cells (Figure 1C) and 53.87 μM in Lovo cells (Figure 1D). Treatment with the respective IC_50_ concentrations of each drug significantly suppressed cell viability in both CRC cell lines (Figure 1E,F). Furthermore, the combination of Baicalin and 5-Fu resulted in a significantly greater reduction in cell viability compared to either agent alone. Furthermore, the inhibitory effect of the combination was significantly greater than the arithmetic sum of the effects of baicalin and 5-Fu alone (*p* < 0.01), indicating a potential beneficial and greater-than-additive interaction, indicating a potential synergistic interaction between the two agents against CRC cells (Figure 1E,F).

### 2.2. Baicalin and 5-Fu Synergistically Inhibit CRC Progression

**Figure 2:**
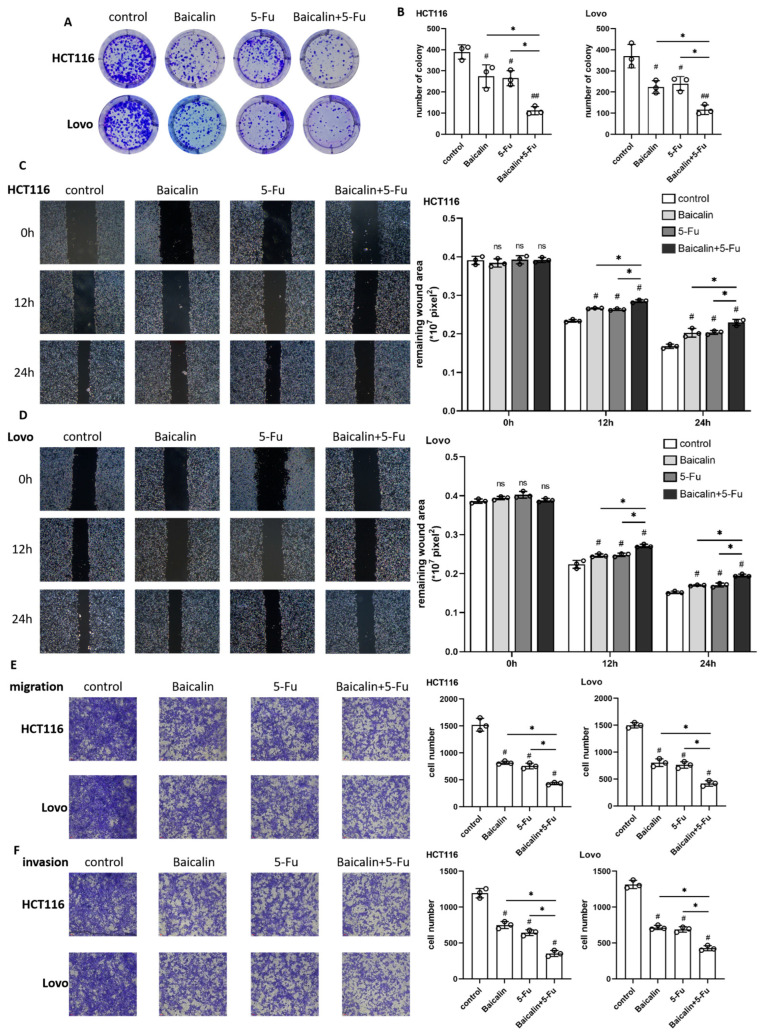
Inhibitory effects of Baicalin on the malignant phenotypes of colorectal cancer cells. (**A**) Colony formation assay results for HCT116 and Lovo cells treated with Baicalin, 5-Fu, or their combination; (**B**) Quantitative analysis of colony formation efficiency corresponding to (**A**); (**C**) Wound healing assay of HCT116 cells under Baicalin, 5-Fu, or combined treatment; (**D**) Wound healing assay of Lovo cells under the same treatment conditions as (**C**); (**E**) Cell migration assay results for HCT116 and Lovo cells after respective treatments; (**F**) Cell invasion assay results for HCT116 and Lovo cells following the indicated treatments (magnification: 200×). Data are expressed as mean ± SEM from three independent biological replicates (n = 3). ns *p* > 0.05, # *p* < 0.05, ## *p* < 0.01 compared to the control group; ns *p* > 0.05, * *p* < 0.05, ** *p* < 0.01, *** *p* < 0.001 for comparisons between treatment groups.

The two cell lines were subjected to four treatment conditions: control, Baicalin monotherapy, 5-Fu monotherapy, and the Baicalin-5-Fu combination. The malignant phenotypes of the cells were then evaluated. Colony formation assays revealed that both monotherapies inhibited CRC cell growth, while the combination treatment exerted a more potent suppressive effect (Figure 2A,B). Wound healing assays, monitored at 0, 12, and 24 h, showed that both drugs effectively hindered the planar migration of CRC cells over time (Figure 2C,D), and the co-administration of Baicalin and 5-Fu consistently resulted in more robust inhibition of cell migration. Transwell migration and invasion assays further confirmed that each drug alone attenuated the migratory and invasive capacities of CRC cells, an inhibitory effect that was markedly enhanced by the combination treatment (Figure 2E,F), and this was not just a simple superposition of the two effects. Collectively, these findings demonstrate that the combination of baicalin and 5-Fu potently suppresses CRC progression colorectal cancer progression.

### 2.3. Baicalin Induces Necroptosis in CRC Cells

**Figure 3:**
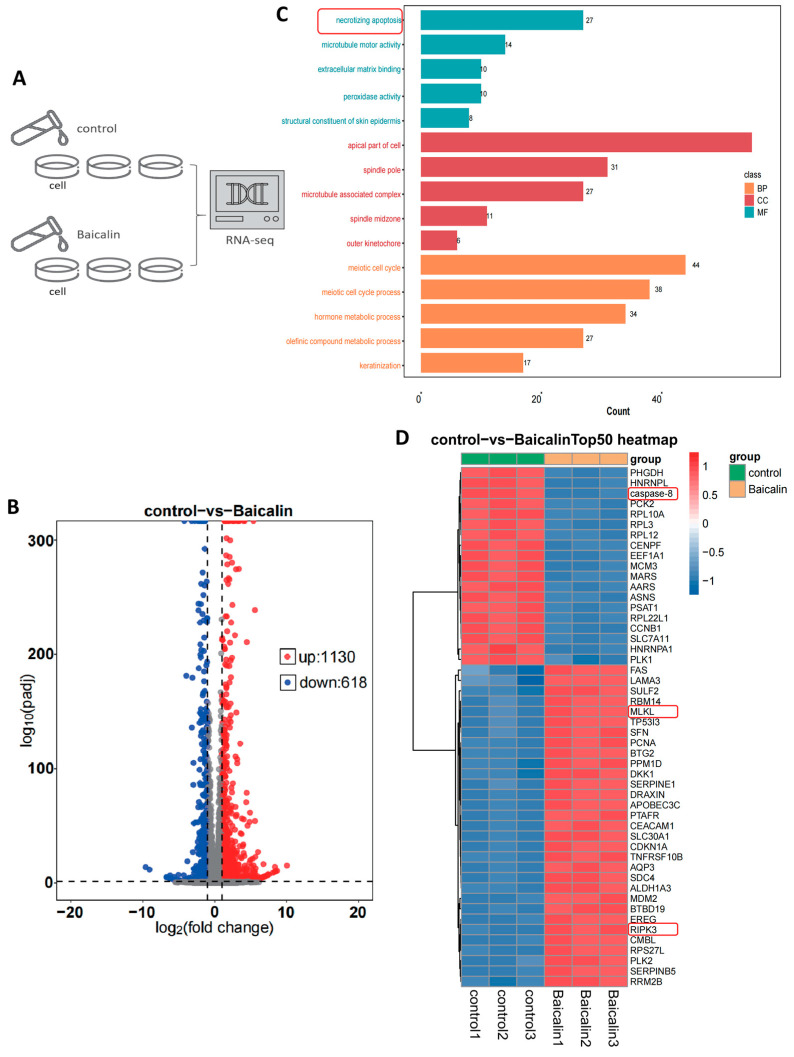
Transcriptomic profiling of colorectal cancer cells treated with Baicalin. (**A**) Schematic diagram of the RNA sequencing experiment conducted on HCT116 cells; (**B**) Volcano plot illustrating differentially expressed genes (DEGs) induced by Baicalin treatment; (**C**) Gene Ontology (GO) pathway enrichment analysis of the identified DEGs; (**D**) Heatmap showing the clustering of the top 50 DEGs between the control and Baicalin-treated groups.

To elucidate the mechanism underlying Baicalin’s suppressive effect on CRC, we performed high-throughput RNA sequencing on Baicalin-treated HCT116 cells and compared their transcriptomic profile to that of untreated controls (Figure 3A). Transcriptomic analysis identified 1748 differentially expressed genes (DEGs), of which 1130 were significantly upregulated and 618 were significantly downregulated (Figure 3B). Gene Ontology (GO) enrichment analysis of these DEGs suggested that the necroptosis pathway is a primary mechanism through which Baicalin exerts its inhibitory effect (Figure 3C). This finding was further supported by a heatmap showing that multiple key genes involved in the necroptosis pathway were ranked among the top 50 DEGs (Figure 3D). Taken together, these results imply that the antitumor activity of Baicalin in CRC is closely linked to its ability to activate the necroptosis pathway.

### 2.4. Baicalin Enhances 5-Fu Efficacy Through Necroptosis Activation in CRC

**Figure 4:**
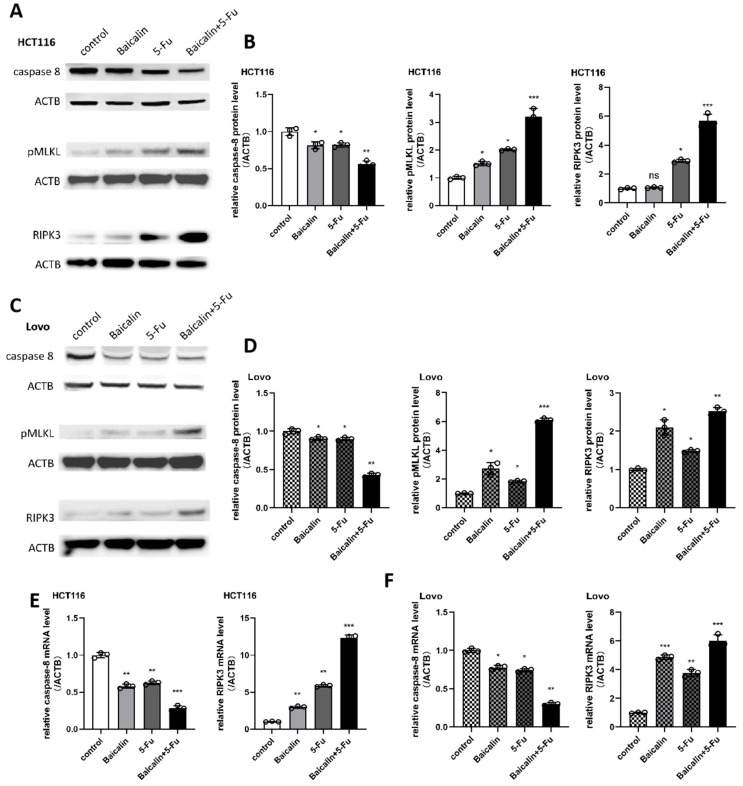
Analysis of key necroptosis-related protein and gene expression levels. (**A**) Western blot results for key proteins in the necroptosis pathway in HCT116 cells; (**B**) Quantitative densitometric analysis of the Western blot bands from (**A**); (**C**) Western blot analysis of necroptosis-related proteins in Lovo cells; (**D**) Quantitative analysis of the Western blot bands from (**C**); (**E**) qRT-PCR results for key necroptosis-related genes in HCT116 cells; (**F**) qRT-PCR analysis of necroptosis-related gene expression in Lovo cells. Data are presented as mean ± SEM from three independent biological replicates (n = 3). ns *p* > 0.05, * *p* < 0.05, ** *p* < 0.01, *** *p* < 0.001 compared to the control group.

To verify the involvement of the necroptosis pathway in the Baicalin-induced suppression of CRC, we examined the expression levels of key necroptosis pathway genes and proteins in the two CRC cell lines. In HCT116 cells, Western blot analysis revealed that the protein level of caspase-8, a well-characterized negative regulator of the necroptosis pathway, decreased following Baicalin treatment. This decrease was even more pronounced when Baicalin was combined with 5-Fu (Figure 4A,B). Conversely, the expression levels of phosphorylated MLKL (pMLKL), the key executioner protein of the necroptosis pathway, and RIPK3, a critical upstream kinase in the pathway, were both found to be upregulated upon Baicalin treatment (Figure 4A,B). Similar expression trends for these proteins were observed in Lovo cells (Figure 4C,D). These protein-level findings were further supported by qPCR analysis, which confirmed consistent alterations in the mRNA expression of these key genes related to necroptosis, in both HCT116 and Lovo cell lines (Figure 4E,F). Notably, treatment with 5-Fu alone also induced similar changes in the expression of these genes, and the effects were markedly enhanced in the combination treatment group. These results collectively suggest that Baicalin likely synergizes with 5-Fu to inhibit CRC progression by activating the necroptosis pathway.

### 2.5. MLKL-Mediated Necroptosis Mediates the Synergistic Effect of Baicalin and 5-Fu in CRC

**Figure 5:**
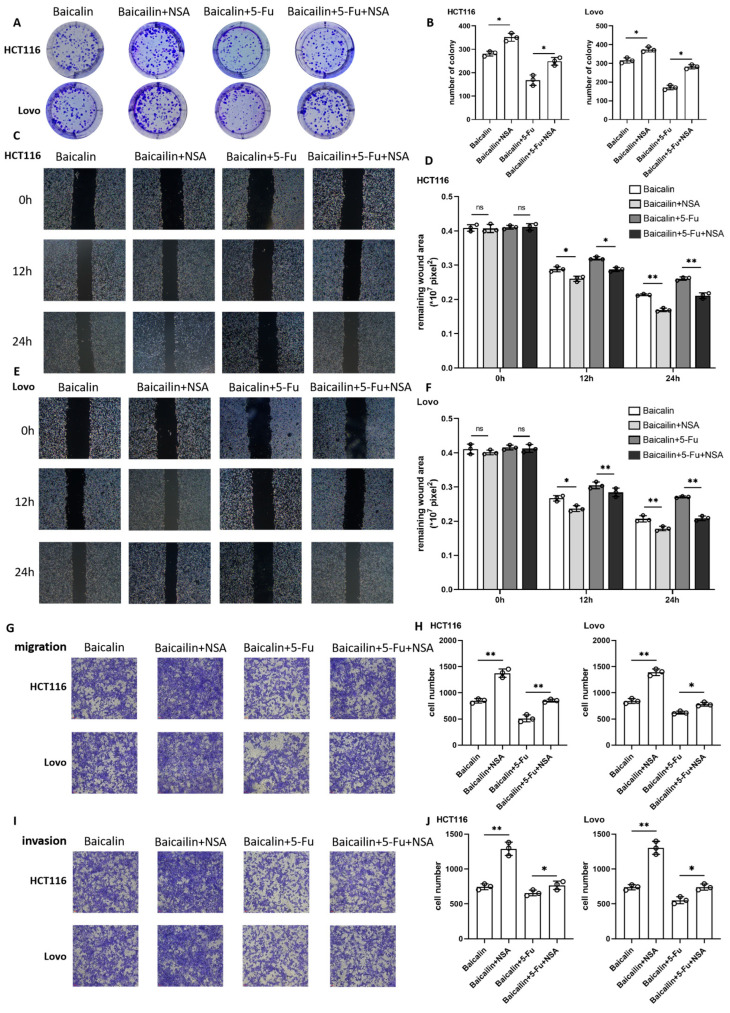
Functional rescue of colorectal cancer cells by inhibiting necroptosis with Necrosulfonamide (NSA). (**A**) Colony formation assay of HCT116 and Lovo cells treated with Baicalin, Baicalin+5-Fu, with or without NSA; (**B**) Quantitative analysis of colony formation from (**A**); (**C**,**E**) Wound healing assays of HCT116 and Lovo cells, respectively, under different treatment conditions; (**D**,**F**) Quantitative analysis of wound closure rates corresponding to (**C**,**E**); (**G**,**I**) Cell migration and invasion assays of HCT116 and Lovo cells following NSA treatment; (**H**,**J**) Quantitative analysis of cell migration and invasion from (**G**,**I**) (magnification: 200×). Data are expressed as mean ± SEM from three independent biological replicates (n = 3). ns *p* > 0.05, # *p* < 0.05, ## *p* < 0.01 compared to the control group; ns *p* > 0.05, * *p* < 0.05, ** *p* < 0.01, *** *p* < 0.001 for comparisons between treatment groups.

To further clarify the contribution of necroptosis to the synergistic cytotoxicity of Baicalin and 5-Fu, we conducted a series of rescue experiments using the MLKL-specific inhibitor Necrosulfonamide (NSA). Cells treated with Baicalin alone or in combination with 5-Fu were concurrently exposed to NSA at its IC_50_ concentration (124 nM). Subsequent phenotypic assays revealed that inhibiting necroptosis partially reversed the drug-induced suppression of CRC. Colony formation assays showed a partial restoration of cell growth upon NSA addition (Figure 5A,B). Similarly, wound healing assays indicated accelerated migration in both cell lines at all observed time points following NSA treatment (Figure 5C–F). Transwell migration and invasion experiments further confirmed that NSA partially rescued the inhibitory effects of Baicalin and 5-Fu (Figure 5G–J). These findings indicate that the MLKL-mediated necroptosis pathway plays a pivotal role in mediating the synergistic anti-CRC effects of Baicalin and 5-Fu.

### 2.6. Inhibition of MLKL-Mediated Necroptosis Attenuates the Antitumor Efficacy of Baicalin In Vivo

**Figure 6:**
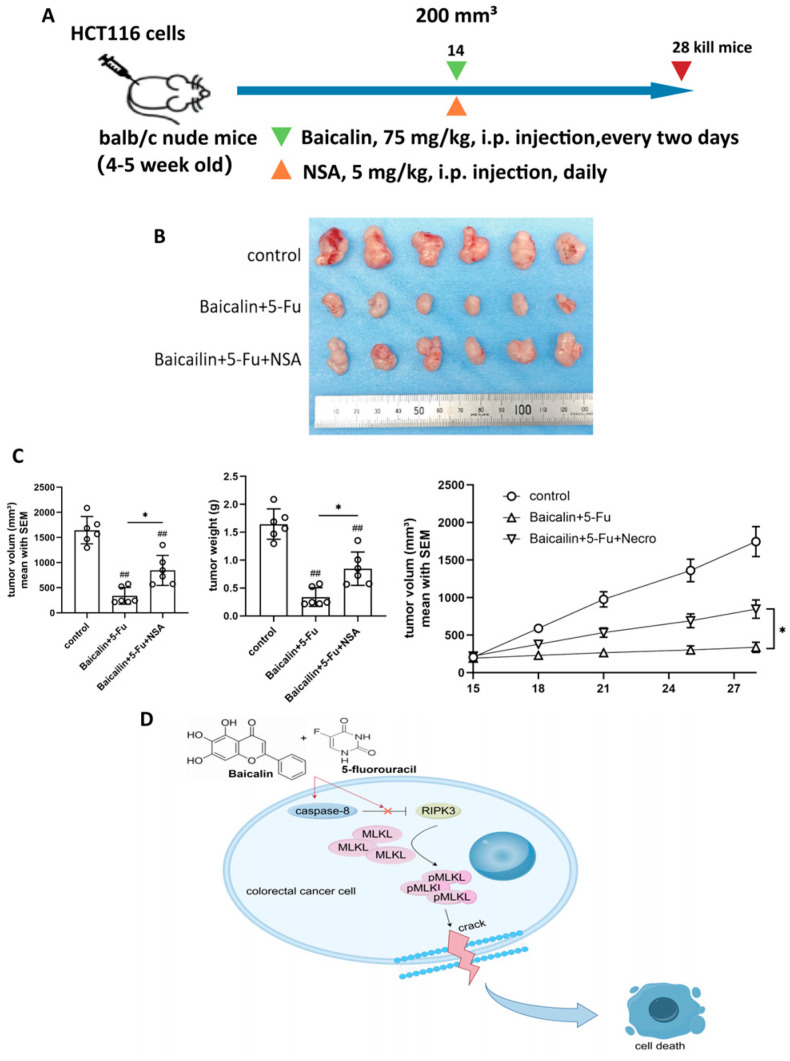
Inhibition of MLKL attenuates the antitumor effect of Baicalin in vivo and schematic model of the synergistic mechanism. (**A**) Schematic diagram of the in vivo rescue experiment using Baicalin and NSA; (**B**) Tumor growth curves of xenograft tumors in nude mice under different treatment conditions; (**C**) Quantitative analysis of tumor weight from (**B**); (**D**) Schematic illustration of the proposed mechanism by which Baicalin synergizes with 5-Fu to induce necroptotic cell death in colorectal cancer cells. Data are presented as mean ± SEM (n = 6). ns *p* > 0.05, # *p* < 0.05, ## *p* < 0.01 compared to the control group; ns *p* > 0.05, * *p* < 0.05, ** *p* < 0.01, *** *p* < 0.001 for comparisons between treatment groups.

To validate these findings in an in vivo setting, we established a xenograft model by subcutaneously inoculating HCT116 cells into nude mice. Once tumor volumes reached approximately 200 mm^3^, mice received Baicalin either alone or in combination with NSA (Figure 6A). After a two-week treatment period, the mice were euthanized, and tumor volume and weight were measured (Figure 6B,C). The results demonstrated that NSA administration partially attenuated the antitumor efficacy of the Baicalin and 5-Fu combination therapy.

Based on the collective findings, a mechanistic model is proposed (Figure 6D): The combination of Baicalin and 5-Fu inhibits caspase-8 expression, which relieves its suppression on RIPK3. This leads to RIPK3-mediated phosphorylation of MLKL (pMLKL). The resulting pMLKL then induces plasma membrane disruption, triggering necroptotic cell death in CRC cells. This mechanism underlies the observed synergistic effect with 5-Fu.

## 3. Discussion

This study systematically demonstrates that Baicalin, a bioactive compound derived from traditional Chinese medicine, potentiates the anticancer activity of 5-Fu to suppress key malignant phenotypes of CRC. Most importantly, we provide the first evidence that this enhanced antitumor effect is primarily mediated through the activation of MLKL-dependent necroptosis, uncovering a novel molecular mechanism underlying Baicalin’s anti-CRC activity and offering a compelling strategy to enhance conventional chemotherapy—particularly in overcoming 5-Fu resistance.

Our investigation began by characterizing the individual and combined cytotoxic effects of both agents. The observed dose-dependent suppression of viability in HCT116 and Lovo cells, along with their distinct IC_50_ values, highlights the inherent heterogeneity in CRC cell responses to chemotherapeutic agents. The significant enhancement of growth inhibition in combination treatments at IC_50_ concentrations strongly suggested a synergistic interaction, a notion robustly confirmed across multiple functional assays. It is important to note that while our data consistently demonstrate a greater-than-additive inhibitory effect of the baicalin/5-Fu combination across multiple functional assays, a formal assessment of enhancement using combination index (CI) analysis was not performed. This improvement needs to be further evaluated in our future experiment. The combination regimen most potently suppressed colony formation, wound closure, and Transwell migration/invasion, indicating that Baicalin potentiates 5-Fu’s efficacy beyond a simple additive effect, presumably by engaging complementary cytotoxic pathways.

To unravel the underlying mechanism, we employed an unbiased transcriptomic approach. RNA sequencing and subsequent GO enrichment analysis clearly pointed to necroptosis as a key pathway activated by Baicalin. This finding holds profound significance in the context of 5-Fu therapy. Necroptosis, a regulated, caspase-independent mode of cell death, serves as an alternative execution pathway when apoptotic mechanisms are impaired [23,24], making it an attractive target for overcoming chemoresistance. Recent studies have shown that reactivating non-apoptotic cell death pathways, such as necroptosis, can effectively sensitize drug-resistant tumor to chemotherapy [25,26], which aligns with our findings.

Our validation experiments consistently demonstrated, both in protein and mRNA levels, Baicalin—either alone or in combination with 5-Fu—downregulates the apoptotic initiator caspase-8 while upregulating the core necroptotic components RIPK3 and phosphorylated MLKL (p-MLKL). Notably, the transcriptional upregulation of MLKL mRNA revealed by RNA-seq was accompanied by a significant increase in p-MLKL protein, while the total MLKL protein level remained largely unchanged. This observation is both coherent and mechanistically informative. It underscores the rapid and efficient nature of necroptotic signaling, wherein existing cellular pools of MLKL protein are swiftly phosphorylated by upstream kinases (e.g., RIPK3) in response to death stimuli, executing cell death before de novo protein synthesis from the upregulated mRNA can appreciably alter the total protein pool. This kinetic hierarchy—where post-translational modification (phosphorylation) precedes and is initially independent of changes in total protein abundance—strongly supports the conclusion that Baicalin actively triggers the necroptosis execution phase.

Interestingly, our previous work in gastric cancer demonstrated that baicalin enhances 5-Fu efficacy by promoting ROS-mediated ferroptosis [17]. In stark contrast, the current study identifies MLKL-dependent necroptosis as the primary synergistic mechanism in CRC. This divergence suggests that the mode of cell death induced by the baicalin/5-Fu combination may be context-dependent, varying by cancer type or even by the specific genetic makeup of the tumor. It underscores the multi-targeted nature of baicalin and highlights the importance of investigating its effects in a tissue-specific manner. This finding opens up new avenues for research into the upstream signals that dictate the switch between ferroptosis and necroptosis in response to this drug combination. This is the first study to identify MLKL-dependent necroptosis as a key mechanism of Baicalin’s action in CRC; it is the first to demonstrate that Baicalin can enhance 5-Fu efficacy specifically by activating this necroptotic pathway; and the combination of transcriptomic discovery, molecular validation, and functional rescue experiments provides a level of mechanistic proof that distinguishes our work from previous descriptive studies.

The inhibition of caspase-8 is a well-established molecular switch that permits necrosome assembly and initiates the necroptotic cascade [27,28]. Subsequent RIPK3-mediated phosphorylation of MLKL leads to its oligomerization and the process of plasma membrane localization, ultimately leading to membrane disruption and lytic cell death [29,30,31]. We therefore propose a mechanistic model wherein Baicalin initiates necroptotic signaling, potentially by suppressing caspase-8 activity, thereby releasing the inhibitory brake on RIPK1/RIPK3 complex formation. Meanwhile, 5-Fu induces DNA damage and apoptotic stress, creating a cellular environment where the activation of this parallel death pathway results in profound synergistic cytotoxicity. Notably, this mechanism may be particularly effective in 5-Fu-resistant CRC cells, which often exhibit reduced apoptotic capacity but retain the ability to undergo necroptosis.

The causal role of MLKL-mediated necroptosis was definitively established through rescue experiments using the specific MLKL inhibitor NSA. The significant, albeit partial, reversal of the combination therapy’s effects on colony formation, migration, and invasion upon NSA treatment provides direct functional evidence that this pathway is indispensable for the observed enhancement. The partial rescue also suggests the potential involvement of additional mechanisms, such as ferroptosis or autophagy, which warrants future investigation. Preliminary in vivo data from xenograft models further corroborated the relevance of this pathway in a more complex physiological setting, reinforcing the translational potential of our findings.

The clinical relevance of our findings is significant, as it directly addresses the pivotal issue of 5-Fu resistance. First, the Baicalin/5-Fu combination presents a promising strategy to enhance therapeutic efficacy while potentially reducing the required dose of 5-Fu, thereby mitigating its dose-limiting toxicities such as myelosuppression and gastrointestinal mucositis—an approach consistent with recent findings on biological compounds [32,33]. This “chemo-sensitization” strategy could improve patients’ quality of life and treatment adherence [34], which are often compromised by 5-Fu’s adverse effects. Second, and most importantly, by activating necroptosis, this combination can effectively target CRC cells that have developed resistance to apoptosis. Recent clinical studies have shown that patients with CRC harboring apoptotic defects have poorer responses to 5-Fu-based chemotherapy [35], and our findings suggest that Baicalin could reverse this resistance by engaging the necroptotic pathway. This offers a potential therapeutic avenue for refractory cancers, where treatment options are currently limited. While our in vivo study utilized a specific dosing schedule, the translation of this combination to the clinic will require careful optimization of administration frequency and sequence. Future pharmacokinetic/pharmacodynamic (PK/PD) studies are essential to determine whether concurrent or sequential administration of baicalin and 5-Fu maximizes necroptotic enhancement while minimizing systemic toxicity. Given Baicalin’s excellent safety profile, it is plausible that a chronic, low-dose metronomic schedule of baicalin could sustainably ‘prime’ tumors for enhanced necroptotic response to subsequent 5-Fu treatment.

Third, the excellent safety profile of Baicalin reported in previous studies [36,37], further enhances its translational potential as an adjuvant. Unlike conventional chemotherapeutic combinations that often add to toxicity burdens, Baicalin’s low systemic toxicity makes it suitable for long-term administration, which may be necessary to overcome persistent drug resistance. Moreover, since necroptosis is an immunogenic cell death modality, its induction may stimulate antitumor immunity [24,38,39], creating a “double hit” against CRC by both directly killing cancer cells and enhancing the host immune response. Recent studies have highlighted the crosstalk between necroptosis and anti-tumor immunity [40,41], which could further improve treatment outcomes and reduce the risk of recurrence—an important consideration in CRC management.

Our study also addresses a critical gap in the field by providing a mechanistic basis for Baicalin’s role in overcoming 5-Fu resistance. While previous studies have reported the antitumor effects of Baicalin in various cancers [16,19], its specific role in reversing 5-Fu resistance in CRC has not been explored. By demonstrating that Baicalin targets MLKL-dependent necroptosis to sensitize CRC cells to 5-Fu, our findings open new avenues for the development of targeted combination therapies. For instance, future studies could explore the potential of combining Baicalin with other necroptosis inducers or immune checkpoint inhibitors to further enhance therapeutic efficacy, particularly in advanced or metastatic CRC.

Notably, our study has several limitations. First, the direct molecular target of Baicalin that initiates the necroptotic cascade remains unidentified. Further studies, such as molecular docking and pull-down assays, are needed to elucidate the specific binding partner of Baicalin and its role in regulating the necroptosis pathway. Second, the use of immunocompromised mouse models precluded assessment of how this combination therapy influences the anti-tumor immune response—a critical aspect of long-term tumor control and overcoming resistance. Future studies using immunocompetent models are necessary to fully evaluate the immunomodulatory effects of the Baicalin/5-Fu combination. While our findings in HCT116 and Lovo cells are robust and provide the first evidence of a novel mechanism, we acknowledge that the use of only two cell lines limits the generalizability of our conclusions regarding the reversal of 5-Fu resistance across the broad spectrum of CRC. These results should be considered a strong foundation for future studies that will validate this synergistic effect and its mechanism in a larger panel of CRC cell lines with well-characterized 5-Fu resistance profiles, as well as in patient-derived organoids. Finally, detailed pharmacokinetic studies and optimized dosing schedules for the combination in vivo are required to guide future clinical development. Taken together, these findings, while preliminary, justify the continued pursuit of the Baicalin/5-Fu combination as a promising new strategy against CRC, including 5-Fu-resistant forms.

In summary, our work not only delineates a novel mechanism whereby Baicalin synergizes with 5-Fu via MLKL-mediated necroptosis but also solidly positions this combination as a viable and promising therapeutic strategy for CRC. By targeting necroptosis, this combination has the potential to overcome 5-Fu resistance—a major clinical obstacle—while minimizing adverse effects. Future research will focus on identifying the direct target of Baicalin, validating the efficacy and immunomodulatory effects in immunocompetent models, and conducting systematic preclinical toxicology and pharmacokinetic studies to pave the way for clinical translation. An important consideration for any anticancer therapy is its selectivity for malignant cells. While our study demonstrates that the baicalin/5-Fu combination effectively induces necroptosis in CRC cells, the effect on normal colonic epithelial cells warrants further investigation. Theoretically, the combination’s reliance on activating an alternative death pathway in apoptosis-resistant cancer cells may offer a degree of tumor selectivity. However, future studies should directly compare the cytotoxic effects of this combination on CRC cells versus normal colon organoids or primary epithelial cells to confirm its therapeutic window and rule out off-target toxicity to healthy tissues.

## 4. Materials and Methods

### 4.1. Cell Culture

We use two human colon cancer cell lines in the study: HCT116 and Lovo, they were obtained from the American Type Culture Collection (ATCC, Manassas, VA, USA). Both cell lines were maintained in High-Glucose DMEM (Servicebio, China, Wuhan, #G4515), supplemented with 10% fetal bovine serum (FBS; Procell, China, Wuhan, #164210) and 1% penicillin-streptomycin-amphotericin B solution (Servicebio, China, Wuhan, #G4015), under 5% CO_2_ at 37 °C.

### 4.2. Reagents and Treatment

Baicalin (purity > 98%; Yuanye, China, Shanghai, #B20570) and 5-fluorouracil (Solarbio, China, Beijing, #F8300) were used in this study. When cells reached approximately 60% confluence, they were exposed to a range of concentrations of the two drugs and incubated for an additional 24 h. Following incubation, cells were harvested via trypsinization and quantified for subsequent experiments. The concentration ratios of Baicalin to 5-fluorouracil are provided in Supplementary Table S1. The following antibodies were used: Caspase 8 Polyclonal antibody (Proteintech, China, Wuhan, #13423-1-AP), Phospho-MLKL antibody (Cell Signaling Technology, Boston, MA, USA, #18640), RIPK3 Polyclonal antibody (Proteintech, China, Wuhan, #17563-1-AP), Beta Actin Monoclonal antibody (Proteintech, China, Wuhan, #66009-1-Ig), Conjugated Goat anti-rabbit IgG Goat Polyclonal Antibody (Huabio, China, Hangzhou, #HA1121: iFluor™ 488), and Conjugated Goat anti-mouse IgG Polyclonal Antibody (Huabio, China, Hangzhou, #HA1125: iFluor™ 488). The MLKL inhibitor Necrosulfonamide (NSA; MedChemExpress, Monmouth Junction, NJ, USA, #1360614-48-7) was also utilized.

### 4.3. CCK8 Assay

Cells were seeded in 96-well plates at a density of 2000 cells per well in 100 µL of medium and cultured for 24 h (37 °C, 5% CO_2_). After drug treatment, 10 µL of Cell Counting Kit-8 (CCK-8; Servicebio, China, Wuhan, #G4103) reagent was added to each well, taking care to avoid bubble formation. Following this, the plates were put back into the incubator for a duration of two hours, and absorbance was measured at 450 nm using a BioTekSynergy microplate reader from the USA, Vermont. The cell viability rate was determined in accordance with the manufacturer’s protocol.

### 4.4. Colony Formation Assay

In conducting the colony formation assay, cells were seeded at a low density of 500 cells per well in six-well plates and treated with the chosen drugs. The plates were cultured for two weeks under standard conditions, with weekly replacement of the medium and drugs. At the conclusion of the incubation period, cells were fixed with 4% paraformaldehyde for 20 min at room temperature. The fixative was then aspirated, and each well was washed three times with phosphate-buffered saline (PBS) to remove residual paraformaldehyde. The fixed cells were stained with a 0.1% crystal violet solution (Servicebio, China, Wuhan, #G1014-50 mL) for 20 min. After staining, the solution was removed, and each well was thoroughly rinsed three times with PBS to eliminate unbound dye. Finally, the stained colonies were documented using a light box.

### 4.5. Wound Healing Assay

To facilitate consistent imaging, reference marks were made on the bottom of the culture plate. When cells reached approximately 80% confluence, a sterile pipette tip was used to make a consistent scratch wound in the cell monolayer. The wells were then gently washed to remove dislodged cells and replenished with a serum-free medium. Cell migration into the wound area was monitored over time, with images captured at predetermined intervals using a microscope.

### 4.6. Transwell Assay

The upper chambers of the Transwell inserts (#3422, Costar, New York, NY, USA) were pre-coated with a thin layer of BD Matrigel for the invasion assay (#356234, CorningNew York, NY, USA), diluted in a serum-free medium, and allowed to solidify. This coating step was omitted when assessing cell migration. Subsequently, identical procedures were followed for both assays. Cells were resuspended in a serum-free medium and seeded into the upper chambers at a density of 1 × 10^5^ cells/well, along with the respective drugs. The lower chambers were filled with a medium containing 10% FBS, which served as a chemoattractant. The plates were incubated for 24 h under standard culture conditions. After incubation, the medium was carefully aspirated from the chambers. The membranes were gently rinsed with PBS to remove non-migratory/non-invasive cells. Cells on the lower surface of the membrane were fixed with 4% paraformaldehyde and subsequently washed with PBS. The fixed cells were stained with 0.1% crystal violet and rinsed thoroughly. Cells remaining on the upper surface of the membrane were carefully removed using cotton swabs. Following air-drying, the membranes were visualized, and cells that had migrated or invaded to the underside were imaged under an inverted microscope at 200× magnification.

### 4.7. Quantitative Real-Time PCR (qRT-PCR)

After 24 h of drug treatment in six-well plates, total RNA was extracted from the cells using the Trizol method (Trizol reagent, Sigma-Aldrich, St. Louis, MO, USA), with trichloromethane used for phase separation. Complementary DNA (cDNA) was synthesized from the purified RNA using a commercial reverse transcription premix (Evo M-MLV, Accurate Biology, China, Hunan, #AG11706). Quantitative PCR (qPCR) amplification was performed using a SYBR Green-based qPCR master mix (Accurate Biology, China, Hunan, #AG11761) with cDNA as the template. The thermocycling conditions included an initial denaturation step (95 °C for 20 s), followed by 40 cycles of denaturation (95 °C for 3 s) and combined annealing/extension (60 °C for 30 s), with a final hold at 25 °C. The nucleotide sequences of the primers used were as follows: caspase-8 forward primer (FP) 5′-GCAAAGGAAGCAAGAACCCAT-3′, reverse primer (RP) 5′-CTGCCTGGTGTCTGAAGTTCC-3′; RIPK3 FP 5′-ATGTCGTGCGTCAAGTTATGG-3′, RP 5′-CGTAGCCCCACTTCCTATGTTG-3′; ACTB FP 5′-GTGACGTTGACATCCGTAAAGA-3′, RP 5′-GCCGGACTCATCGTACTCC-3′.

### 4.8. Western Blot Analysis

Following PBS washes, cells were lysed directly in their culture dishes. The resulting lysates were centrifuged, and the clarified supernatants were collected for protein concentration determination using a BCA assay kit (Epizyme, China, Nanjing, #ZJ101). Equal amounts of protein from each sample were separated by electrophoresis on 15% SDS-polyacrylamide gels and subsequently transferred to nitrocellulose membranes. The membranes were blocked with 5% skim milk prepared in TBST for one hour at room temperature, followed by three washes with TBST. The membranes were then probed with specific primary antibodies in a cold room overnight. After another series of TBST washes, the membranes were incubated with appropriate secondary antibodies for one hour at room temperature. Immunoreactive protein bands were detected and visualized using a near-infrared dual-color fluorescence imaging system (Licor, Odyssey CLx, Lincoln, NE, USA).

### 4.9. RNA Sequencing and Bioinformatic Analysis

Total RNA extracted from the various treatment groups underwent high-throughput sequencing on an Illumina platform (conducted by Apexbio Company, Houston, TX, USA). Differential gene expression analysis between Baicalin-treated and control groups was performed, with genes meeting the criteria of |log_2_ fold change (FC)| > 1 and an adjusted *p*-value < 0.05 defined as differentially expressed genes (DEGs). Gene Ontology (GO) enrichment analysis was subsequently performed on the identified DEGs, and the results were graphically represented using R-4.2.3 software.

### 4.10. In Vivo Xenograft Tumor Model

Specific pathogen-free (SPF) BALB/c nude mice, aged 5–6 weeks, were randomly assigned to different experimental groups. A suspension of HCT116 human colorectal cancer cells (1 × 10^7^ cells in 100 µL) was subcutaneously inoculated into the flank of each mouse. After a six-week period of tumor growth, the mice were humanely euthanized, and the resulting subcutaneous tumors were carefully excised. Tumor volume and weight were recorded. Tumor volume (V) was calculated using the formula V = (L × W^2^)/2, where L and W correspond to the longest and perpendicular shortest tumor diameters, respectively. The doses of Baicalin (50 mg/kg) and 5-Fu (20 mg/kg) were used in our xenograft models. Baicalin and NSA were administered daily via intraperitoneal injection due to their relatively short half-lives, while 5-Fu was given twice weekly to mimic clinical cycles and reduce cumulative toxicity. Treatment was initiated when tumors reached approximately 200 mm^3^ to model established disease.

### 4.11. Statistical Analysis

Data were statistically analyzed using ordinary one-way analysis of variance (ANOVA) combined with Tukey’s post hoc test for multiple group comparisons. A probability (*p*) value of less than 0.05 was considered statistically significant. Detailed statistical parameters are included in the Supplementary Table S1. ImageJ-win 64 (Fiji) software was used for tasks including cell counting, quantification of mean fluorescence intensity, and densitometric analysis of protein bands. Visualizations such as volcano plots, heatmaps, and GO enrichment plots were generated using R software. All data graphs and charts were constructed using GraphPad Prism 8 software.

## 5. Conclusions

In conclusion, this study demonstrates that Baicalin synergizes with 5-Fu to suppress CRC progression in vitro and in vivo. Mechanistically, we uncover that this synergistic effect is primarily mediated through the activation of MLKL-dependent necroptosis, a pathway distinct from the ferroptosis mechanism previously observed in gastric cancer. Our findings not only reveal a novel antitumor mechanism of baicalin but also provide a strong scientific rationale for repurposing this well-tolerated natural compound as a promising adjuvant to enhance 5-Fu efficacy and potentially overcome chemoresistance in colorectal cancer. This work lays a solid foundation for future preclinical development and ultimate clinical evaluation of this combination strategy.

## Data Availability

The corresponding authors will provide the datasets supporting this study upon reasonable request.

## Supplementary Materials

The following supporting information can be downloaded at: https://www.mdpi.com/article/10.3390/ijms27062919/s1.

## References

[1] Bray F., Laversanne M., Sung H., Ferlay J., Siegel R.L., Soerjomataram I., Jemal A. (2024). Global cancer statistics 2022: GLOBOCAN estimates of incidence and mortality worldwide for 36 cancers in 185 countries. CA A Cancer J. Clin..

[2] Biller L.H., Schrag D. (2021). Diagnosis and Treatment of Metastatic Colorectal Cancer: A Review. JAMA.

[3] Morris V.K., Kennedy E.B., Baxter N.N., Benson A.B., Cercek A., Cho M., Ciombor K.K., Cremolini C., Davis A., Deming D.A. (2022). Treatment of Metastatic Colorectal Cancer: ASCO Guideline. J. Clin. Oncol..

[4] Shin A.E., Giancotti F.G., Rustgi A.K. (2023). Metastatic colorectal cancer: Mechanisms and emerging therapeutics. Trends Pharmacol. Sci..

[5] Vodenkova S., Buchler T., Cervena K., Veskrnova V., Vodicka P., Vymetalkova V. (2019). 5-fluorouracil and other fluoropyrimidines in colorectal cancer: Past, present and future. Pharmacol. Ther..

[6] Argilés G., Tabernero J., Labianca R., Hochhauser D., Salazar R., Iveson T., Laurent-Puig P., Quirke P., Yoshino T., Taieb J. (2020). Localised colon cancer: ESMO Clinical Practice Guidelines for diagnosis, treatment and follow-up. Ann. Oncol..

[7] Yang L., Yang J., Kleppe A., Danielsen H.E., Kerr D.J. (2023). Personalizing adjuvant therapy for patients with colorectal cancer. Nat. Rev. Clin. Oncol..

[8] Singh U., Kokkanti R.R., Patnaik S. (2025). Beyond chemotherapy: Exploring 5-FU resistance and stemness in colorectal cancer. Eur. J. Pharmacol..

[9] Xie S., Zhou Z., Zheng Y., Yu C., Kong W., Chen Y., Si W., Zhou F., Yang Z., Ni R. (2025). Novel drug research and therapeutic strategies targeting tumor metastasis and cancer stem cells. Front. Pharmacol..

[10] Alzahrani S.M., Al Doghaither H.A., Al-Ghafari A.B., Pushparaj P.N. (2023). 5-Fluorouracil and capecitabine therapies for the treatment of colorectal cancer (Review). Oncol. Rep..

[11] Das T., Anand U., Pandey S.K., Ashby C.R., Assaraf Y.G., Chen Z.-S., Dey A. (2021). Therapeutic strategies to overcome taxane resistance in cancer. Drug Resist. Updates.

[12] Kleszcz R., Majchrzak-Celińska A., Baer-Dubowska W. (2023). Tannins in cancer prevention and therapy. Br. J. Pharmacol..

[13] Tan Y.-Q., Lin F., Ding Y.-K., Dai S., Liang Y.-X., Zhang Y.-S., Li J., Chen H.-W. (2022). Pharmacological properties of total flavonoids in *Scutellaria baicalensis* for the treatment of cardiovascular diseases. Phytomedicine.

[14] Kong N., Chen X., Feng J., Duan T., Liu S., Sun X., Chen P., Pan T., Yan L., Jin T. (2021). Baicalin induces ferroptosis in bladder cancer cells by downregulating FTH1. Acta Pharm. Sin. B.

[15] Gao Q., Sheng Q., Yang Z., Zhu Z., Li L., Xu L., Xia J., Qiao Y., Gu J., Zhu X. (2025). Honokiol-Magnolol-Baicalin Possesses Synergistic Anticancer Potential and Enhances the Efficacy of Anti-PD-1 Immunotherapy in Colorectal Cancer by Triggering GSDME-Dependent Pyroptosis. Adv. Sci..

[16] Hu Q., Zhang W., Wu Z., Tian X., Xiang J., Li L., Li Z., Peng X., Wei S., Ma X. (2021). Baicalin and the liver-gut system: Pharmacological bases explaining its therapeutic effects. Pharmacol. Res..

[17] Yuan J., Khan S.U., Yan J., Lu J., Yang C., Tong Q. (2023). Baicalin enhances the efficacy of 5-Fluorouracil in gastric cancer by promoting ROS-mediated ferroptosis. Biomed. Pharmacother..

[18] Wu Y., Fang Y., Li Y., Au R., Cheng C., Li W., Xu F., Cui Y., Zhu L., Shen H. (2023). A network pharmacology approach and experimental validation to investigate the anticancer mechanism of Qi-Qin-Hu-Chang formula against colitis-associated colorectal cancer through induction of apoptosis via JNK/p38 MAPK signaling pathway. J. Ethnopharmacol..

[19] Jia Q., Zhou Y., Song L., Shi X., Jiang X., Tao R., Wang A., Wu Y., Wei Z., Zhang Y. (2024). Baicalin reduces chronic stress-induced breast cancer metastasis via directly targeting β2-adrenergic receptor. J. Pharm. Anal..

[20] Yang Y., Hu Q., Shao Q., Peng Y., Yu B., Luo F., Chen J., Xu C., Li Z., Tam M. (2025). A Baicalin-Based Functional Polymer in Dynamic Reversible Networks Alleviates Osteoarthritis by Cellular Interactions. Adv. Sci..

[21] Liu F., Meng F., Hong X., Giri A.K., Asim M.H., Chen Z., Chen Y., Lin Y., He L., Bu Q. (2025). Preparation and evaluation of Baicalin-loaded albumin nanoparticles for anti-breast cancer activity. Int. J. Biol. Macromol..

[22] Zhang H., Yang X., Zhao L., Jiao Y., Liu J., Zhai G. (2015). In vitro and in vivo study of Baicalin-loaded mixed micelles for oral delivery. Drug Deliv..

[23] Meier P., Legrand A.J., Adam D., Silke J. (2024). Immunogenic cell death in cancer: Targeting necroptosis to induce antitumour immunity. Nat. Rev. Cancer.

[24] Tang R., Xu J., Zhang B., Liu J., Liang C., Hua J., Meng Q., Yu X., Shi S. (2020). Ferroptosis, necroptosis, and pyroptosis in anticancer immunity. J. Hematol. Oncol..

[25] Grassilli E., Narloch R., Federzoni E., Ianzano L., Pisano F., Giovannoni R., Romano G., Masiero L., Leone B.E., Bonin S. (2013). Inhibition of GSK3B bypass drug resistance of p53-null colon carcinomas by enabling necroptosis in response to chemotherapy. Clin. Cancer Res..

[26] Nicolai S., Pieraccioli M., Peschiaroli A., Melino G., Raschellà G. (2015). Neuroblastoma: Oncogenic mechanisms and therapeutic exploitation of necroptosis. Cell Death Dis..

[27] Yuan J., Ofengeim D. (2023). A guide to cell death pathways. Nat. Rev. Mol. Cell Biol..

[28] Ai Y., Meng Y., Yan B., Zhou Q., Wang X. (2024). The biochemical pathways of apoptotic, necroptotic, pyroptotic, and ferroptotic cell death. Mol. Cell.

[29] Yuan F., Cai J., Wu J., Tang Y., Zhao K., Liang F., Li F., Yang X., He Z., Billiar T.R. (2022). Z-DNA binding protein 1 promotes heatstroke-induced cell death. Science.

[30] Pasparakis M., Vandenabeele P. (2015). Necroptosis and its role in inflammation. Nature.

[31] Lawlor K.E., Murphy J.M., Vince J.E. (2024). Gasdermin and MLKL necrotic cell death effectors: Signaling and diseases. Immunity.

[32] Newton K., Wickliffe K.E., Dugger D.L., Maltzman A., Roose-Girma M., Dohse M., Kőműves L., Webster J.D., Dixit V.M. (2019). Cleavage of RIPK1 by caspase-8 is crucial for limiting apoptosis and necroptosis. Nature.

[33] Pettersen C.H.H., Samdal H., Sætrom P., Wibe A., Hermansen E., Schønberg S.A. (2023). The Salmon Oil OmeGo Reduces Viability of Colorectal Cancer Cells and Potentiates the Anti-Cancer Effect of 5-FU. Mar. Drugs.

[34] Li H., Wang B., Wang Y. (2022). 2′-Fucosyllactose Suppresses Angiogenesis and Alleviates Toxic Effects of 5-Fu in a HCT116 Colon Tumor-Bearing Model. Molecules.

[35] Su Z., Liu M., Krohn M., Schwarz S., Linnebacher M. (2025). The impact of SEC23A on 5-FU chemotherapy sensitivity and its involvement in endoplasmic reticulum stress-induced apoptosis in colorectal cancer. Apoptosis.

[36] Geng J., Zhang Y., Gao Q., Neumann K., Dong H., Porter H., Potter M., Ren H., Argyle D., Bradley M. (2021). Switching on prodrugs using radiotherapy. Nat. Chem..

[37] Jiang P., Chipurupalli S., Yoo B.H., Liu X., Rosen K.V. (2025). Inactivation of necroptosis-promoting protein MLKL creates a therapeutic vulnerability in colorectal cancer cells. Cell Death Dis..

[38] Wang L., Feng T., Su Z., Pi C., Wei Y., Zhao L. (2022). Latest research progress on anticancer effect of baicalin and its aglycone baicalein. Arch. Pharm. Res..

[39] Bailly C. (2023). Efficacy and safety of the traditional herbal medication Chai-Ling-Tang (in China), Siryung-tang (in Republic of Korea) or Sairei-To (in Japan). J. Ethnopharmacol..

[40] Hänggi K., Li J., Gangadharan A., Liu X., Celias D.P., Osunmakinde O., Keske A., Davis J., Ahmad F., Giron A. (2024). Interleukin-1α release during necrotic-like cell death generates myeloid-driven immunosuppression that restricts anti-tumor immunity. Cancer Cell.

[41] Rucker A.J., Park C.S., Li Q.J., Moseman E.A., Chan F.K.-M. (2024). Necroptosis stimulates interferon-mediated protective anti-tumor immunity. Cell Death Dis..

