# Supplementary material for: Baicalin Augments 5-Fluorouracil Efficacy in Colorectal Cancer by Triggering MLKL-Dependent Necroptosis: A Novel Strategy to Overcome Chemoresistance
Source: Int J Mol Sci. 2026 Mar 23;27(6):2919. doi: 10.3390/ijms27062919 (PMC13026119; doi:10.3390/ijms27062919)
Supplement: Supplementary file 1 [file ijms-27-02919-s001.zip › ijms-4159286-supplementary.pdf]

Table S1. Concentrations of Baicalin and 5-Fu used for single-agent IC_{50} determination. ("weight" refers to the weight of the drug in its dry powder form, "concentration (μM)" refers to the concentration of the drug after it is mixed evenly with 1 ml of deionized sterile water).

Drug	Weight (μg)	Concentration (μM)
Baicalin	30	67.21
	60	134.42
	120	268.84
	240	537.68
	480	1075.37
5-Fu	1.7	5
	3.4	10
	6.8	20
	13.6	40
